# Risk factor for breast cancer development under exposure to bovine leukemia virus in Colombian women: A case-control study

**DOI:** 10.1371/journal.pone.0257492

**Published:** 2021-09-21

**Authors:** Nury N. Olaya-Galán, Sandra P. Salas-Cárdenas, Jorge L. Rodriguez-Sarmiento, Milcíades Ibáñez-Pinilla, Ricardo Monroy, Adriana P. Corredor-Figueroa, Wilson Rubiano, Jairo de la Peña, HuaMin Shen, Gertrude C. Buehring, Manuel A. Patarroyo, Maria F. Gutierrez

**Affiliations:** 1 PhD Program in Biomedical and Biological Sciences, Universidad del Rosario, Bogotá, Colombia; 2 Grupo de Enfermedades Infecciosas, Laboratorio de Virología, Departamento de Microbiología, Pontificia Universidad Javeriana, Bogotá, Colombia; 3 Department of Pathology, Hospital Universitario San Ignacio - Pontificia Universidad Javeriana, Bogotá, Colombia; 4 Hospital Universitario Mayor Méderi – Universidad del Rosario, Bogotá, Colombia; 5 School of Public Health, University of California, Berkeley, California, United States of America; 6 Molecular Biology and Immunology Department, Fundación Instituto de Inmunología de Colombia (FIDIC), Bogotá, Colombia; 7 Microbiology Department, Faculty of Medicine, Universidad Nacional de Colombia, Bogotá, Colombia; 8 Health Sciences Division, Main Campus, Universidad Santo Tomás, Bogotá, Colombia; Consejo Nacional de Investigaciones Cientificas y Tecnicas, ARGENTINA

## Abstract

Viruses have been implicated in cancer development in both humans and animals. The role of viruses in cancer is typically to initiate cellular transformation through cellular DNA damage, although specific mechanisms remain unknown. Silent and long-term viral infections need to be present, in order to initiate cancer disease. In efforts to establish a causative role of viruses, first is needed to demonstrate the strength and consistency of associations in different populations. The aim of this study was to determine the association of bovine leukemia virus (BLV), a causative agent of leukemia in cattle, with breast cancer and its biomarkers used as prognosis of the severity of the disease (Ki67, HER2, hormonal receptors) in Colombian women. An unmatched, observational case–control study was conducted among women undergoing breast surgery between 2016–2018. Malignant samples (n = 75) were considered as cases and benign samples (n = 83) as controls. Nested-liquid PCR, *in-situ* PCR and immunohistochemistry were used for viral detection in blood and breast tissues. For the risk assessment, only BLV positive samples from breast tissues were included in the analysis. BLV was higher in cases group (61.3%) compared with controls (48.2%), with a statistically significant association between the virus and breast cancer in the unconditional logistic regression (adjusted-OR = 2.450,95%CI:1.088–5.517, *p* = 0.031). In this study, BLV was found in both blood and breast tissues of participants and an association between breast cancer and the virus was confirmed in Colombia, as an intermediate risk factor.

## 1 Introduction

Cancer represents one of the greatest threats to public health worldwide. It is responsible for about 163.5 deaths per 100,000 inhabitants per year and is considered one of the most common causes of death, that is highly related with a lower life expectancy. In females, breast and cervical cancers are the two most commonly etiologies worldwide [1, 2]. Although high incidence rates have decreased in high-income countries, it continues to increase in other regions such as South America, with a rapid burden of the disease that has led to a peak of breast cancer in the last ten years [3, 4]. In Colombia, breast cancer is the main cancer etiology among women, with 13,380 new cases reported in 2018 and a mortality rate of 12.0 per 100,000 inhabitants [5].

Viruses have been proposed in the literature as potential starters of cellular transformation and tumorigenesis in both humans and animals [6, 7]. About 15–20% of cancers are correlated with a virus infection that leads to cellular transformation and tumorigenesis processes, with different mechanisms reported among them that are involved in the initial stages of cancer development [8]. Human papilloma virus, human herpes virus 8, Epstein-Barr virus, and hepatitis B and C virus are well-known examples of viruses associated with cancer in humans. Tumorigenesis is usually described as a slow process, that could take decades after the initial infection for the final outcome, in which viral infections could remain quiescent, latent or at a very low viral load in the host for several years until cancer development [6, 9]. Some of the mechanisms associated with cancer development are involved in epigenetic and genomic factors, such as the accumulation of mutations, inhibition of DNA repair mechanisms, induction of host genome instability, degradation of p53 in host cells and chronic inflammatory processes [10, 11].

Considering cancer as a multifactorial disease, the identification of external factors that may be associated with the development of cancer, including viruses, opens an alternative to introduce prevention and control strategies to reduce the risk of cancer [12]. However, there are still gaps in the knowledge in terms of cancer causation. Bradford-Hill criteria support the theory of causation of external factors related with cancer and suggests causality [13] as in the case of HPV with cervical cancer when fulfilling stated criteria [14]. Thus, in order to establish if a particular virus could be considered as a causative agent of cancer, the first necessary step is to perform studies from the epidemiological point of view. These studies should be focused on the identification of viral agents in specific cancer cells, with consistency along different geographical regions and populations, allowing the research community to identify potential risk factors associated with cancer development.

Regarding breast cancer, some viral agents have been reported in previous studies as potential risk factors of the disease [15]. Among them, Epstein-Barr virus (EBV), human papillomavirus (HPV), mouse mammary tumor virus (MMTV) and bovine leukemia virus (BLV) have been identified in human breast tissues [16]. It is important to note that no conclusive evidence has yet been found with respect to the causative relationship between the viral agents and the breast cancer development. However, it has been hypothesized that those viruses could be present on the breast tissues could be involved in the initiation of tumorigenesis and tissue transformation [17].

BLV is an exogenous deltaretrovirus (*Retroviridae* family), the causative agent of chronic infections in cattle, leading to leukemia and/or lymphoma development in between 5–10% of infected animals [18]. Unlike other retroviruses, deltaretroviruses (e.g. HTLV, STLV and BLV) cause malignant transformation mainly through a multifaceted protein (Tax) involved in several processes of regulation of the host cell [19, 20]. Tumorigenesis mediated by these viruses occurs several years after infection, in which the viruses remain integrated into the host cells, with no or low evidence of the viral infection and without clinical manifestations.

In previous studies, BLV biomarkers (e.g. genes’ fragments, antibodies and proteins) have been reported to be present in women [21–25]. In addition, the association of BLV with breast cancer has been suggested in case-control studies performed in USA, Brazil and Australia with Odd Ratios (ORs) ranging between 2.7 and 5.0, with significant *p* values for each specific population, proposing BLV as an intermediate risk factor for breast cancer development [26–28]. However, those results are inconclusive in terms of considering the virus a causative agent of breast cancer, as other studies have reported contradictory findings [29, 30]. In Argentina, significant results were obtained in the comparison between the presence of the virus with breast cancer prognostic markers such as Ki67 (cell division marker) and HER-2 (epidermal growth factor), suggesting that BLV might be involved in severity and progression of the disease, favoring cellular proliferation [31]. In Brazil, analysis of the tumor markers compared with the presence of BLV was performed, although no statistical differences were identified [28].

Breast cancer profile is determined by the presence/absence of specific tumor markers located on the surface of the cells, such as hormonal receptors (i.e. estrogens (ER) and progesterone (PR)), overexpression of HER-2 protein, and cell division marker Ki67 [32]. Together, these are the basis for categorizing breast cancer in terms of severity, progression of the disease and are useful for personalized treatment alternatives depending on the hormonal and tumor markers profile, that represent more than 21 subtypes of breast cancer [33]. Luminal A is the most prevalent subtype, which includes patients with positive hormonal receptors but negative HER-2 and it is considered a cancer subtype with slow-growing rate and good prognosis for recovery. In contrast, triple negative cancer subtype (HER-2 (-), ER (-) and PR (-)) represents a worst prognosis in the patients, as no specific treatment is available for this type of cancer with a high-rate of cellular proliferation. Few studies are available in terms of comparing cancer subtypes with other exogenous risk factors as viruses, focused on cancer progression and severity [34].

Molecular epidemiology studies in human populations focused on assessing BLV as a risk factor for cancer development are essential for clarifying the role of this virus in breast cancer and other cancer types, considering the oncogenic potential of other deltaretroviruses such as HTLV. This study was consequently aimed at determining the association between BLV and breast cancer in Colombian patients, as well as its correlation with progression tumor markers and cancer subtypes. BLV was identified as an intermediate risk factor in the analyzed population in line with other regions around the world.

## 2 Materials and methods

### 2.1 Study design

An unmatched, observational case–control study was designed for determining the association of the presence of BLV with breast cancer in a population of Colombian women between 2016 and 2018. Participants were women with breast tumors, benefited from the breast surgical service at Méderi Hospital (MH) located in Bogotá, Colombia. Following the histopathological diagnosis (see below, section 2.3) and the clinical records of the patients, participants were divided into two groups (i.e., cases and controls).

Cases were defined as patients diagnosed with any type of breast cancer, whilst patients diagnosed with benign pathology of the breast were considered as the control group, which was used as a reference for further analyses. The study was approved by the ethics committee of Universidad del Rosario (UR) and Méderi Hospital (Record No. CEI-ABN026-000 241, 2016). All procedures were performed in accordance with the ethical standards of the institution and with the 1964 Helsinki declaration and its later amendments (last revision 2013). All the participants voluntarily signed an informed consent prior to the surgical procedures. Data obtained during the study was used under confidentiality.

From the patients benefited with the surgical intervention, the inclusion criteria were: women patients over 18 years of age, with a minimum tumor size of 4mm and enough biological material for both pathology follow-up and BLV detection. As exclusion criteria, samples with high content of fat tissue or low quality for molecular biology were discarded. Other diseases were not considered as exclusion criteria.

Sample size was calculated with a post-hoc strategy for the obtained OR of 2.45, with a case: control relation of 75:83, showing a power of 80% (type II error– 20%) and a confidence interval of 95% (type I error– 5%). Sample size was not possible to determine in advance, due to the lack of previous evidence in 2016 in Latin America and Colombia. A single study was published, reporting the presence of BLV in Colombian women [22]. None of the participants withdrew from the study.

### 2.2 Study variables

For the analyses of the results, exposure factor (independent variable) was defined by the presence of molecular markers of BLV, which was a dichotomous nominal variable categorized as positive or negative regarding the results obtained from the molecular biology techniques. Pathologies of the breast were considered as the dependent dichotomous nominal variables, defined as cases when categorized as malignant breast tissues, and as controls for benign breast tissues.

In addition, sociodemographic characteristics were obtained from the patients: age, educational level, city of origin, occupation, family background of cancer and parity history. Also, the complete clinical records of the patients were available, from which biomarkers of breast cancer used for the prognosis of the disease were obtained (hormonal receptors, Ki67, HER2) and sociodemographic variables were confirmed.

As confounding variables were considered: age, parities, background of cancer in the family and educational level. Only age was considered as a quantitative variable and was recategorized in groups ≥50 and <50, regarding to the risk factor group for breast cancer [35]. Confounding variables were used to adjust the model in further analyses.

### 2.3 Data and samples collection

After the inform consent was signed, participants answered a survey prior surgery, in order to collect the information of the sociodemographic variables mentioned above. Samples were collected consecutively and sequentially between 2016 and 2018 from patients who were scheduled for breast surgery in MH. Blood and breast tissue were taken from each patient for the study. Fresh breast tissue was placed in new, empty, sterile flasks and immediately transported to the virology lab at Pontificia Universidad Javeriana (PUJ). Blood samples were drawn into EDTA anticoagulant tubes and were also taken to PUJ.

### 2.4 Samples’ preparation and pathological diagnosis

Collected breast samples were used for both histopathological diagnosis and viral detection. One section of the tissue was formalin-fixed in 10% formalin buffer and embedded in paraffin (FFPE) for histopathological classification in the San Ignacio University Hospital (HUSI), following the World Health Organization (WHO) international standards [36], approved protocol by the College of American Pathologists (CAP) for invasive cancer resection [37]. Pathology results were confirmed with MH’s clinical records, and cases and controls were identified.

The second tissue section, as well as blood samples, were used for gDNA extraction with a High Pure PCR Template Preparation Kit (Roche ^®^, Mannheim, Germany), following the manufacturer’s instructions. From blood, mononuclear cells were recovered with Lymphosep reagent ^®^ (MP, Solon, OH—USA). Extracted DNA was stored at -20ºC until use.

### 2.5 Bovine leukemia virus detection

#### 2.5.1 Nested-liquid phase PCR (nPCR)

Quality of the extracted DNA from blood and breast samples was validated by amplifying the human GAPDH housekeeping gene. GAPDH-positive samples were used in further analyses. BLV detection targeted BLV genome regions (*gag*, *LTR*, *tax* and *env*). Primers and PCR cycling conditions from a previous report [21] were used here, with slight adjustments to PCR cycle conditions. Roche’s PCR Master Mix (Cat. No. 11636103001, Mannheim, Germany) and Promega’s GoTaq polymerase (Madison, WI—USA) were used for detection. Two researchers confirmed the results separately (NOG at UC Berkeley and SSC at PUJ), with an accuracy of 90%. The results were visualized by gel electrophoresis on 1.5% agarose gels stained with ethidium bromide. DNA extracted from the FLK cell line (constitutively infected with BLV) was used as positive control. As a negative control of reaction, RNAse/DNAse free water–molecular grade was used in each experiment. As an internal control of the laboratory, DNA from MCF7 (human breast cancer) cell line, which is negative to BLV, was used for discarding contamination of the areas and is included randomly in the experiments to avoid the presence of false positive results. Also, for avoiding crossed contamination, separate hoods were used for master mix preparation, DNA samples addition, and positive control addition to the PCR reaction. Samples were considered positive when at least one of the virus’s genes was amplified and confirmed by Sanger sequencing, to ensure that it was a BLV product.

#### 2.5.2 Direct *in situ* PCR (IS PCR)

Direct *in situ* PCR was used as secondary test for viral detection in FFPE breast tissue, as previously described [21]. Slight changes were performed to the PCR protocol. From the FFPE tissues, extra cuts were performed and were attached to SuperFrost slides (Thermo Fisher ^™^, Hayward–CA, USA), as suggested by Nuovo [38]. The technique was optimized by targeting a longer region of the *tax* gene (nt 7197–7570, F:CTTCGGGATCCATTACCTGA; R:GCTCGAAGGGGGAAAGTGAA, 373bp). After paraffin removal, tissues were digested with pepsin (2mg/mL) with 0.05mL 2N HCl. *In situ* PCR was performed with the digoxigenin-labelled uracil system (Roche, Mannheim, Germany) and AmpliTaq Gold DNA polymerase—hot-start (Applied Biosystems ^™^, Carlsbad–CA, USA). Reactions were detected by an anti-DIG monoclonal antibody (mAb) (Roche, Mannheim, Germany) and revealed with DAB (diaminobenizidine) solution, following the manufacturer’s instructions (Vector ^®^, Burlingame–CA, USA). An adjacent tissue section from each sample, without Taq polymerase and without primers, was evaluated to verify that no cross-reaction or non-specific attachment occurred by the DIG-labelled uracil and/or by the mAb as a negative reaction control. FLK cell line smears were used as positive controls. Slides were observed under a Nikon Eclipse E200, at 10x/40x magnification. Samples were considered positive when a dark brown-red stain was visualized in the mammary epithelial cells (ducts and lobules), and were clearly differentiated from the background. Negative control tissue displayed no brown color.

#### 2.5.3 Viral proteins detection by immunohistochemistry

Viral capsid protein (p24) was detected by immunohistochemistry (IHC) on an extra slide of breast tissue. Endogenous peroxidases were inactivated with 3% hydrogen peroxide in methanol solution, followed by unmasking antigens in citrate buffer (10mM sodium citrate buffer, pH 6.0) in boiling water (95ºC) for 30 min. Tissues were blocked with 1.5% fetal horse serum (FHS) in PBS preventing non-specific antibody attachment. A mAb targeting p24 diluted 1/10 in blocking solution and a biotinylated horse anti-mouse IgG secondary antibody (1/200) (Vector Laboratories Cat# BP-2000, RRID:AB_2687893) were used for viral detection. An ABC kit (Vector ^®^, Burlingame–CA, USA) was used as reaction enhancer and the DAB reagent (Vector ^®^, USA) was used for peroxidase activity detection. Results were observed on a Nikon Eclipse E200 optical microscope at 10x/40x magnification. Dark brown coloring in mammary epithelial cells was considered positive, representing p24 in the cells. As a negative control, an adjacent tissue section was treated only with the secondary antibody.

For the statistical analyses for the risk assessment, presence of BLV in the breast tissues of the patients was considered positive when at least one of the PCR techniques (nested-liquid PCR or *in situ* PCR) was able to identify the viral DNA and was confirmed by sequencing. Correlation of the presence of the virus in breast and blood was also evaluated.

### 2.6 Cancer prognosis biomarkers and hormonal receptors

Information regarding the tumors’ hormone profile (progesterone receptors–PR and estrogen receptors–ER) and prognostic markers (HER2 and Ki67) were retrieved from the cancer patients’ clinical records. Tests were performed by the pathology diagnosis laboratory of Méderi Hospital, following internal protocols. Immunohistochemistry was performed by pre-diluted monoclonal antibodies directed to the specific markers from Dako/Agilent ^®^ (Santa Clara—CA, USA). HER2 protein was detected with HercepTest (Cat.No.SK00121-5), rabbit anti-human monoclonal antibody (Agilent Cat# IR084, RRID:AB_2617140); progesterone receptors (PR) with mouse anti-human monoclonal antibody (Agilent Cat# IR06861, clone PgR636, RRID:AB_2890066); estrogen receptors (ER) with rabbit anti-human monoclonal antibody (Agilent Cat# IR084, clone EP1 RRID:AB_2617140) and Ki67 was detected with mouse anti-human monoclonal antibody (Agilent Cat#IR62661-2CN, cloneMIB-1, RRID:AB_2890068). Results were visualized on an optical microscope Olympus BX43 and results were reported as part of the findings given to the patient in the histopathological report. Tests were not performed to patients with pathologies other than breast cancer.

### 2.7 Principles of comparability, validity and reliability

Defined principles of comparability for analytical case-control studies were used to avoid bias and assure validity in our study [39–41]. For example, the selection of cases and controls form the same basis population, and the control of other risk factors described for breast cancer in the literature (e.g., age, nulliparity, family background of breast cancer and educational level) [33] in the multivariate analysis fulfilling the deconfounding principle and obtaining adjusted OR values.

All variables and samples were measured and processed identically, and by blinded investigators, avoiding measuring bias and having no differences in the manipulation between cases and controls samples guaranteeing the comparable accuracy principle. Classification bias of dependent variable (breast tissue histopathological diagnosis) was controlled by following the CAP protocol for breast cancer diagnosis [37] and was verified by the clinical records at MH. For BLV detection, samples were determined as positive when at least one of the molecular techniques (nested-liquid PCR or *in situ* PCR) showed positive for viral DNA in the breast, as direct evidence of the presence of BLV in the tissue. PCR products were sequenced by Sanger technology confirming identity >95% with BLV.

### 2.8 Statistical analysis

SPSS (Ver. 25.0, IBM Corp., Amonk, NY, USA) and STATA (Ver. 15, StataCorp LP, College Station, TX, USA) were used for statistical analysis. Initially, a descriptive analysis was carried out to all the qualitative variables, from which frequencies and percentages were determined. Measurements of central tendency and dispersion (e.g., average, range, and standard deviation) were used for ‘age’, which was the only quantitative variable. Normality was measured by Shapiro Wilk and Kolmogorov Smirnov tests.

According to the expected values in contingency table (<5), Pearson chi-square or exact Fisher’s test were used for comparing cases and controls variables, as well as cancer prognostic biomarkers (hormonal receptors, Ki67 and HER2) with the presence of the virus. The association between BLV and breast cancer was carried out by an unconditional multivariate logistic regression for the estimation of the Odd Ratios(OR) with a 95% of confidence interval (CI) adjusted by risk factors associated with breast cancer and other confounding variables identified in the study (i.e., age, parity, background of breast and ovarian cancer, educational level, occupation, and city of origin). The prediction area and its respective 95% CI were determined with the ROC curve. *p* values. Less than 0.05 were considered as significant for the study (*p* < 0.05) for all the statistical analyses). As a secondary analysis, pre-malignant samples diagnosed in the histopathological observations were included in the data set, and a multinomial logistic regression was carried out, due to the risk of these pathologies for breast cancer development in the future [42].

## 3 Results

### 3.1 Population’s sociodemographic and biological characteristics

This cohort of patients, obtained between 2016 and 2018, was constituted by a total of 168 participants. After the histopathological classification, 75 patients were diagnosed with cancer (malignant tumors) and were included into the cases group; 83 patients were diagnosed with benign pathologies of the breast and were included in the control group. Ten of the patients were diagnosed with pre-malignant lesions of the breast (i.e., hyperplasia with atypia, *in situ* carcinoma). Those were excluded from the initial analysis, resulting in a definitive cohort of 158 patients.

Participants were aged between 18 and 89 years, and lived in Bogota city, where Méderi Hospital is located. Statistically significant differences were identified between cases and controls in terms of age, educational level, parities, and occupation in the bivariate analysis (See Table 1). In the cases group, patients were older compared with the control group. In addition, cases group had a lower educational level compared with the control group, as well as the occupations reported, which were more frequent to be on the home-basis in the cases group. Sociodemographic characteristics could be influenced by geographical regions and cultural behaviors. In the analyzed population, educational level and occupation were significant for the model, and were included in the multivariate analysis.

**Table 1:** Comparison of sociodemographic and biological characteristics between malignant (cases) and benign samples (controls).

	Cases	Controls	
	Malignant (n = 75)	Benign (n = 83)	*P value*
	n (%)	n (%)	
*Age*			
$\bar{\text{x}}\pm \text{SD}$	66.15 ± 11.89	40.55 ± 18.01	<0.001
≥50	47 (62.7)	10 (12.0)	
Other characteristics			
*Origin*			0.047
Bogotá	61 (81.3)	56 (67.5)	
Other	14 (18.7)	27 (32.5)	
Family background of breast/ ovarian cancer	46 (63.9)	48 (58.5)	0.304
Parity	64 (86.5)	48 (58.5)	<0.001
*Educational level*			<0.001
Elementary school	31 (41.9)	11 (13.4)	
High School	26 (35.1)	28 (34.1)	
Vocational and professional studies	17 (23.0)	43 (52.4)	
*Occupation*			<0.001
Home-based activities	38 (50.7)	15 (18.1)	
Living/working in rural areas	1 (1.3)	1 (1.2)	
Other ^a^	36 (48.0)	67 (80.7)	

^a^ Other: industry, office, own business, marketing, etc.

### 3.2 Histopathological classification and viral detection

Frequencies of the most relevant breast’s pathologies for the cases and control groups were included in both groups. Malignant tumors (cases) were described as invasive ductal carcinoma, invasive lobular carcinoma, and other malignancies (i.e., malignant phyllodes tumor, sarcoma, mixed type carcinoma and invasive poorly differentiated carcinoma). Within the control group, benign pathologies were diagnosed as fibroadenomas, hyperplasia without atypia, papillary lesions, and others less frequent (i.e., simple cysts, benign phyllodes tumor, mastitis). Frequencies of each pathology are found on Table 2 and details of the complete data set can be found in S1 Table in S1 File.

**Table 2:** Histopathological diagnoses and viral detection.

Histopathological diagnoses	BLV DETECTION/TECHNIQUE §					BLV (+) ** *n (%)*
	Nested PCR		IS PCR *n (%)*	nPCR + IS PCR# *n (%)*	IHC (p24) *n (%)*	
	Breast tissue *n (%)*	Blood *n (%)*				
**Cases (n = 75)**						46 (61.3)
Invasive ductal carcinoma (n = 37)	15 (40.5)	13 (35.1)	9 (24.3)	3 (8.1)	4 (10.8)	
Invasive lobular carcinoma (n = 10)	6 (60.0)	5 (50.0)	5 (50.0)	3 (30.0)	2 (20.0)	
Other malignancies ^a^ (n = 28)	12 (42.9)	10 (38.5)	7 (25.0)	4 (14.3)	3 (10.7)	
**Controls (n = 83)**						40 (48.2)
Fibroadenoma (n = 43)	16 (37.2)	12 (28.6)	11 (25.6)	8 (18.6)	3 (6.9)	
Hyperplasia without atypia (n = 10)	2 (20.0)	1 (10.0)	1 (10.0)	1 (10.0)	1 (11.1)	
Papillary lesions (n = 11)	6 (54.5)	3 (27.3)	3 (27.3)	3 (27.3)	2 (22.2)	
Other benign tumors ^b^ (n = 19)	6 (31.6)	4 (22.2)	4 (21.16)	4 (21.1)	3 (10.0)	

^a^Other malignancies: malignant phyllodes tumor (n = 1), sarcoma (n = 1), mixed type carcinoma (lobular and ductal, n = 3), invasive poorly differentiated carcinoma (n = 20), mucinous carcinoma (n = 3).

^b^Other benign tumors: Simple cysts (n = 3), benign phyllodes tumor (n = 1), mastitis (n = 1), fibrocystic change (n = 7), sclerosis adenosis (n = 7).

^**§**^ Results are shown as frequencies per each diagnosis. Percentages were calculated for each specific diagnosis within cases and controls.

^#^nPCR+IS PCR indicate results for samples that were simultaneously positive for both techniques.

** BLV (+) represents the total amount of positive samples for cases and controls.

Table 2 also shows results for the viral detection reported by each technique that was performed. Results are shown by the presence of the virus in blood and breast tissues, organized by the cases and controls groups as well as for each specific histopathological diagnosis. Details of the complete data set could be found in the supplementary material. Each molecular technique was carried out for detecting different targets of the virus (i.e., viral genome segments and viral proteins). The use of different techniques contributes to the understanding of the biological implications of the virus and strengthens the validation of the diagnosis. Results considered for the risk assessment of the association of breast cancer with the presence of the virus, were those in which proviral DNA of BLV was found by the molecular techniques and confirmed by sequencing on the breast samples. S1 and S2 Figs in S1 File show results of nested and *in situ* PCR targeting *gag* and *tax* region respectively. Samples that showed positive to both techniques (nPCR + IS PCR, Table 2) indicated that more than one genetic region of the virus was identified in the same sample. Detection of BLV in the blood and immunohistochemistry were performed for a better understanding of the biological implications of the presence of the virus in human beings. BLV was detected both in blood and breast tissues with a correlation of 94% in the positive samples of the study. IHC results indicate the presence of viral proteins (p24) in the breast tissues. For this study, only 10% of the samples showed the presence of p24 proteins. No significant statistical differences were identified regarding BLV detection among the specific histopathological diagnosis of the cases and controls samples. Detection techniques were directed to the proviral stage of the virus, which remains for long terms in the host. No active viral infection was evaluated. Presence of p24 proteins suggest evidence of complete viral particles in the tissues, besides the evidence of its proviral genome.

### 3.3 Association between presence of BLV and breast cancer

**Fig 1:**
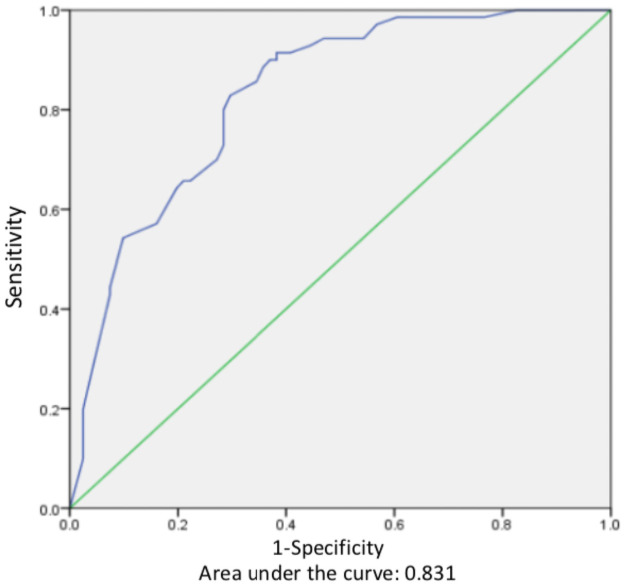
ROC curve of the presence of BLV model predictive of breast cancer.

BLV was found in 61.3% (n = 46) of patients with cancer (cases) and in 48.2% (n = 40) of the control group, being with a higher presence in the cases group. Results obtained in the unconditional logistic regression showed that presence of BLV was significantly associated with breast cancer outcome, compared with the benign pathologies of the breast (OR = 2.45, CI 95%: 1.088–5.517, *p* = 0.031, Table 3), after adjusting with confounding variables including age, parities, background of breast and ovarian cancer in the family, educational level, and occupation. The model prediction area using the ROC curve was significant, with 83.1% (95% CI 76.7%-89.5%, *p* < 0.001, Fig 1). ROC model showed a sensitivity of 77.1%, specificity of 71.6% and accuracy of 74.2%.

**Table 3:** Unconditional logistic regression adjusted by risk factors for breast cancer (age, parities, background of cancer in the family and educational level).

Variables	Malignant (n = 75)		
	ß	OR (95% CI)	*P value*
*Viral presence*			
BLV POS	0.902	2.450 (1.088–5.517)*	0.031*
BLV NEG	--	1.00 (Reference)	--
*Age*			
≥50	2.104	8.202 (3.163–21.270)	<0.001
<50	--	1.00 (Reference)	--
*Nulliparity*			
Yes	-0.714	0.490 (0.174–1.380)	0.177
No	--	1.00 (Reference)	--
*Family background of breast/ovarian cancer*			
Yes	0.140	1.151 (0.499–2.655)	0.742
No	--	1.00 (Reference)	--
*Education level*			0.06
Elementary school	1.155	3.176 (1.114–9.051)	0.031
High School	0.473	1.604 (0.611–4.209)	0.337
Vocational and Professional studies	--	1.00 (Reference)	--

* Significant results obtained for the presence of the virus in the breast cancer population (malignant–cases) compared with benign samples as the reference (control) group. <0.05 *p* values were considered statistically significant for the study.

OR-adjusted values.

### 3.4 Complementary analysis for pre-malignant samples

As the association of BLV with breast cancer was identified in the cohort of patients, pre-malignant samples obtained in the histopathological diagnosis were also included for a secondary analysis. Some of the pre-malignant diagnoses in the breast are considered as precursory lesions of breast cancer, increasing the risk of cancer outcome (e.g. *in situ* carcinomas) [42].

In our study, ten of the patients of the initial cohort were diagnosed with pre-malignant lesions of the breast, distributed as follows: atypical hyperplasia (n = 3), *in situ* carcinoma (n = 4), papillary lesion with atypia (n = 2) and atypical phyllodes tumor (n = 1). These patients were aged between 24–81 years, with a mid-age of 59.60 ± 17.49 years. In terms of educational level, occupation and city of origin, patients were evenly distributed.

Although pre-malignant lesions were not considered initially in the study design, within the cohort of patients ten of them were confirmed as pre-malignant after surgery; and considering the risk of pre-malignant lesions to evolve into breast cancer, and the natural history of the disease, we evaluated if the presence of the virus also influenced the OR in patients with this diagnosis as a complement to the initial analysis, also in terms of not losing valuable information obtained from the study.

A multinomial logistic regression was performed including the pre-malignant samples in the model; thus, breast cancer (cases, n = 75) and pre-malignant samples (n = 10) were compared with benign samples as the reference group. Model was also adjusted with the confounding variables, and a 95% of confidence interval.

In the multinomial logistic regression, an association between the presence of the virus and breast cancer was also identified (Adjusted OR 2.477, 95% CI 1.108–5.538, *p* = 0.027). However, presence of BLV in the pre-malignant lesions did not show a significant association, although OR value was greater than 1.0 (OR 1.133,95% CI: 0.286–4.488, p = 0.859).

### 3.5 Correlation between BLV presence and tumor prognostic markers

When comparing presence of BLV with breast cancer tumor prognostic markers (hormonal receptors, HER2 protein and Ki67), no statistically significant differences were found in the bivariate analysis. This analysis was carried out to determine if the virus could have any implications in the aggressiveness and prognosis of breast cancer. Although no significant differences were identified, a high percentage of BLV positive samples were also positive for estrogen (63%) and progesterone (61%) receptors (Table 4). Bivariate analysis was performed only to the malignant group (n = 75), compared with the viral presence, due to the use of those markers as prognostic markers of breast cancer and are not performed routinely to all the specimens.

**Table 4:** Correlation between hormone receptors and prognostic markers for breast tumors with BLV presence.

TUMOR BIOLOGY	BLV PRESENCE		*P* value
	BLV POS n (%)	BLV NEG n (%)	
***Progesterone Receptors—PR (n = 76)***			0.373
POS (n = 59)	36 (61.0)	23 (39.0)	
NEG (n = 17)	9 (52.9)	8 (47.1)	
***Estrogen Receptors—ER (n = 76)***			
POS (n = 67)	42 (62.7)	25 (37.3)	0.094
NEG (n = 9)	3 (33.3)	6 (66.7)	
***Cellular division marker—Ki67 (n = 68)*** *			
>14 (n = 39)	20 (51.3)	19 (48.7)	0.112
≤14 (n = 29)	20 (69.0)	9 (31.0)	
***Epidermal growth factor—HER2 (n = 69)*** *			
POS (n = 15)	7 (46.7)	8 (53.3)	0.239
NEG (n = 54)	33 (61.1)	21 (38.9)	

*Tests were not performed to all the patients.

## 4 Discussion

Viruses are considered potential initiators of cancerous diseases. Classically, HPV and HBV have been studied for their association with cancer in humans among other viruses as well [9]. However, investigating the role viruses could have in cancer development helps to advance in the current knowledge about cancer etiology. In the future, prevention and control strategies could be implemented to reduce the risk of exogenous risk factors leading to cancer diseases [43]. In this study, an association of the presence of BLV with breast cancer was identified with an OR = 2.45, CI 95%: 1.088–5.517 and a significant *p* value (*p* = 0.031). According to the American Cancer Society (ACS), this OR value represents an intermediate risk factor (ORs 2.0–4.0) for cancer development, along with other risk factors such as radiation, hormonal exposure and having a history of relatives suffering breast/ovarian cancer [33].

In previous studies, higher OR values were identified supporting the association between BLV and breast cancer in other regions like Australia (4.72 OR, 1.71–12.0 95%CI) [27] and the USA (3.07 OR, 1.66–5.69 95%CI) [26]. Nevertheless, differences between the obtained OR values with respect to the presence of the virus in humans could be influenced by sociodemographic conditions of the evaluated populations. The most relevant ones are their ethnic profiles, cultural behaviors, economical income and lifestyle habits, including food consumption. In contrast, the OR value obtained for Colombia was quite similar to that obtained in South Brazil (2.73 OR, 1.18–6.29 95%CI) [28]. Both populations have similar conditions in terms of sociodemographic characteristics and genetic history, involving native American, African and South European ancestry [44, 45]. On the other hand, USA and Australia have greater influence from Northern Europe and Asian countries [26, 46]. Taken together, viral presence and ethnicity could be factors involved in breast cancer outcomes [47]. Regarding the socioeconomical factors, conditions such as accessibility to health insurance, late diagnosis of the disease, resources for management of the disease and lifestyle could also affect these populations, causing higher rates of breast cancer as it has been shown in the literature [48, 49].

Additionally, considering that cattle are naturally infected by the virus, and high prevalence rates have been reported worldwide [50], another possibility of differences among the OR values in those regions could be related with the intake of cattle-derived food products. USA and Australia are classified by the FAO (Food and Agriculture Organization of the United Nations) as high meat and milk consumption countries, while Latin American countries are considered intermediate consumers [51, 52]. The presence of BLV DNA as a biomarker of viral presence in cattle-derived food products was recently reported in Colombia [53]. Therefore, in spite of having no evidence of infectious viral particles, the presence of viral DNA supports the hypothesis of transmission through consuming infected food products, probably through a cell-to-cell infection mechanism [54]. However, further studies are needed to fully understand the virus’s transmission pathway to humans.

Previous studies in Colombia have shown evidence of the virus in women [22, 23], but have not determined whether BLV could be a risk factor for the Colombian population or if it has any implications in the progression of the disease. Results obtained in the current study support the hypothesis of BLV being associated with breast cancer as reported in other regions, but nosignificant difference between the presence of the virus, specific histopathological diagnosis nor the tumor prognosis markers were not found (Table 4). Results showed that BLV could be present in different profiles of the mammary epithelial cells, including tumor profiles and diagnoses. In contrast, results obtained in Argentina [31] showed correlation between BLV and prognosis markers in the breast tumors of women in Tandil.

Analyzing the relationship between breast cancer biomarkers (i.e., hormonal receptors, Ki67 and HER2) and the viral presence, it might give a background of the tumor microenvironment. Itcould be favoring an active viral transcription stage and a specific subtype of breast cancer that is probably associated with BLV infection. Previous *in vitro* studies have shown higher BLV transcription rates induced by progesterone and corticoids stimulation through LTR region activity [55]. In Argentina, a significant correlation between BLV and the Ki67 biomarker was found [31], suggesting that BLV might be involved in early stages of cancer development, as this biomarker indicates an active cell division and proliferation stage of breast cancer. Even if in our study non-significant results were obtained when comparing tumor markers with the presence of BLV, it is important to highlight that most of the samples that were positive for the virus were also positive for hormonal receptors (n = 36 for ER and n = 42 for PR). Moreover, most of the lobular cancer subtype samples (6 out of 10) were positive to the virus. Even if no statistical correlation was possible to obtain due to a small amount of samples with this specific diagnosis, it is important to consider in the future studies of BLV analyses regarding the subclassification of breast cancer, in order to identify if it could be associated to specific subtypes.

Another key point in the current study, was the evidence of BLV in both breast tissues and blood of Colombian women with a concordance of 94%. BLV has been reported in breast tissues [21, 22] and blood [24, 25], but not in the same target population. Evidence of the virus in both blood and breast tissues from the same patient supports the hypothesis that blood might be helping to spread the virus throughout the body, until it reaches other tissues such as breast and lungs, in which the virus has been described to date [56]. Also, there is a possibility that the virus could be reaching other tissues as well, that have not yet been studied, and might interact with permissive cells mediated through the cellular receptors, which are proposed to be AP3D1 [57] or CAT1/SLC7A1 [58].

Now, taking into account the BLV biomarkers that were identified, it is important to underline that in this virus some fragments of its viral genome could be lost after infection [59, 60]. Therefore, revising the presence of the virus with different markers decreases the chances of false negative samples. Moreover, sequencing also guarantees that the amplified products belong to BLV and not to unspecific amplifications. It is important to highlight that the majority of the positive samples showed positive for at least two biomarkers, mainly for the detection in blood and breast (94% of concordance) (S1 Table in S1 File). PCR results indicate presence of the virus in proviral stage (integrated in the host cell genome), while IHC indicates presence of viral proteins, as evidence of active viral replication. In samples in which p24 was identified, other viral markers were also found as expected. Bearing in mind the biology of viruses involved in cancer development, previous evidence in the literature suggests that cancer manifestations could appear several years after initial infection and is not necessary to have an active viral cycle, with the production of new viral particles to induce cellular transformation processes [6].

One of the evaluated markers was the presence of a fragment of *Tax* region within the breast tissues in the *in-situ* PCR. Finding this biomarker, might be associated with cellular transformation, as it happens to cattle and humans in the leukemia development in the case of BLV and HTLV respectively [19, 61]. Tax protein is described as a multifaceted protein which has the capacity of co- regulate different cellular and viral pathways. It acts as a transactivator, inhibits mechanisms of DNA repair and also regulates proliferation and apoptosis pathways, even in few amounts of the protein [62]. However, it remains unclear the specific mechanism or role that BLV might have in humans, besides its association with breast cancer [26–28]. In our study, it was not possible to detect *tax* region with the *in situ* PCR in all of the positive samples, although sequences were confirmed for those cases targeted to *gag* region. Previous evidence in the literature reported for BLV and HTLV suggests that the viruses are not always integrated completely, with the evidence of genomic deletions in natural infection. Nevertheless, it is not clear the implications of these deletions to the viral cycle, as it has been found both in asymptomatic individuals, as well as in advanced stages of cancer disease [60, 63]. Further studies are needed to make clear the functionality of BLV in humans, as well as its integrations profiles to elucidate a plausible role for cancer outcome.

Besides the analysis performed for cases and controls, a secondary analysis involving the pre-malignant samples was considered. These samples were not intended to be in the initial design but were incidental findings of the study. We are conscious it was a few number of samples (n = 10), but considering the risk for cancer development with these lesions [42], it was interesting to observe if the virus could have any impact on these samples as well. In the multinomial logistic regression, the association between BLV and breast cancer was also confirmed with an adjusted OR 2.477, 95% CI 1.108–5.538, *p* = 0.027. Whereas no significant results were obtained when analyzing the association between the presence of the virus and the pre-malignant samples. Further studies with this specific diagnosis are highly recommended to evaluate the impact of BLV in these samples and potential cancer outcome.

Prospective studies with human participants in cancer research are challenging. Obtaining matched-samples for case-control studies, in this case from malignant and benign tumors of the breast, takes long terms due to the availability of surgeries and interventions. In contrast, retrospective studies open the possibility of obtaining higher number of samples from archives, although the quality of the samples is not always indicated for molecular analysis and missing data from the participants is common. An advantage of collecting samples directly from the surgeries provides better quality of breast tissues for DNA processing and availability of blood, as well as the availability of the data collected directly from the participants, which enriches the epidemiological studies. In some cases, the complete clinical records are not available in archive samples.

In our study we performed a design with high complexity in the conception, as well as the detection of the virus through multiple techniques to guarantee processes of validity and comparability. Although paired samples were not taken, the multivariate analysis was controlled with confounding variables described in the literature and was adjusted by age. However, it is important to highlight that even if confusion for the analysis was reduced, it is possible to still have other variables that were not possible to control, leading to a residual confusion for the analysis. Nevertheless, statistical differences were obtained in the study between the cases and controls group, supporting the hypothesis of BLV being associated with breast cancer, contributing to the research field of the role of BLV in humans.

## 5 Conclusion

In conclusion, this study showed the association of BLV with breast cancer in the analyzed population, with an OR value similar to that obtained in Brazil. BLV could be considered as an intermediate risk factor for breast cancer, although further studies are needed to elucidate the role and mechanisms of the virus in humans. Evidence of BLV both in blood and breast tissues, suggests a possibility for early detection of the virus in screening studies.

This study is an incremental finding for the current situation of BLV in humans and its association with breast cancer. Prevention and control strategies of BLV in cattle could favor to stop the transmission of the virus to humans. Eradication programs worldwide should be considered, as it has already been done with eradication policies in Europe, Australia, and New Zealand.

## Supporting information

S1 File Representative results of nested and *in situ* PCR. Participants’ histopathological diagnosis and viral detection.
(PDF)

S1 Raw images (PDF)
