# Supplementary material for: Risk factor for breast cancer development under exposure to bovine leukemia virus in Colombian women: A case-control study
Source: PLoS One. 2021 Sep 21;16(9):e0257492. doi: 10.1371/journal.pone.0257492 (PMC8454960; doi:10.1371/journal.pone.0257492)
Supplement: S1 File — Participants’ histopathological diagnosis and viral detection. (PDF) [file pone.0257492.s001.pdf]

Journal: PLOS ONE

Title: Risk factor for breast cancer development under exposure to Bovine Leukemia Virus in Colombian women: A case-control study

Authors: Nury N. Olaya-Galán[1,2]*, Sandra P. Salas-Cárdenas[2], Jorge L. Rodriguez-Sarmiento[3], Milcíades Ibáñez-Pinilla[4], Ricardo Monroy[4], Adriana P. Corredor-Figueroa[2], Wilson Rubiano[4], Jairo de la Peña[4], HuaMin Shen[5], Gertrude C. Buehring[5], Manuel A. Patarroyo [6,7,8], Maria F. Gutierrez[2]

***Corresponding author:**
nury.olaya@urosario.edu.co (NOG)

S1_File: Representative results of nested and *in situ* PCR. Participants' histopathological diagnosis and viral detection.

Supporting information

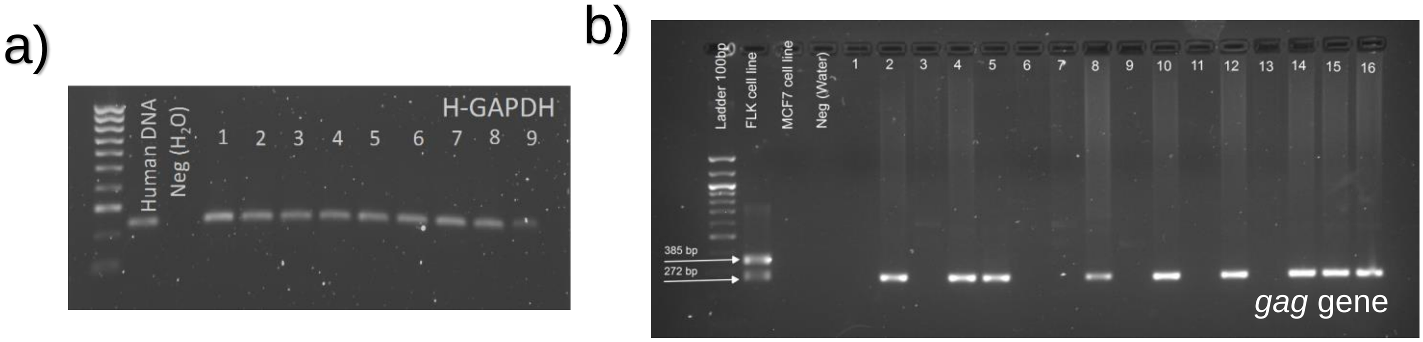

S1 Fig. Liquid-phase nested PCR. Representative agarose gel (1.5%) of human breast tissues and blood samples. Ethidium bromide staining. **(a)** Human GAPDH (H-GAPDH) housekeeping gene. DNA quality verification after extraction. Lanes 1-9: Representative human samples. **(b)** Nested PCR targeting BLV *gag* gene. FLK cell line: positive control, BLV infected - foetal lamb kidney cell line. MCF7 cell line: breast cancer cell line (ATCC – HTB-22), negative amplification control. Neg (water): negative reaction control. Lanes 1-16: Representative human samples positive and negative for BLV. *Results for H-GAPDH and *gag* gene were visualized in two different electrophoresis gels. No changes in color and contrast were performed to the gels.

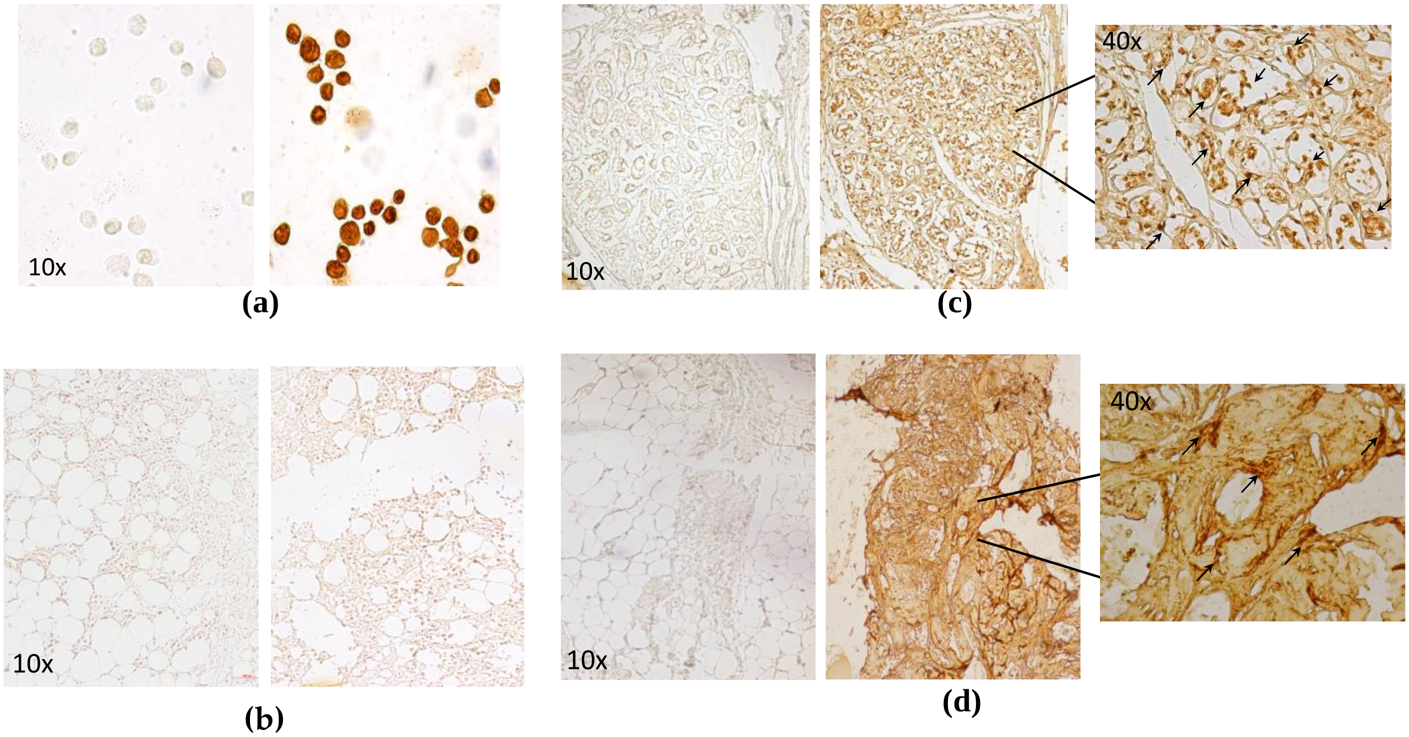

S2 Fig. *In situ* PCR of human breast tissue. *Left panels* – Adjacent tissue slice/smear for antibody control. *In situ* PCR reaction without primer and Taq polymerase control. *Right panel*s – Complete *in situ* PCR reaction. Staining was visualised by DAB reaction against anti-DIG system. **(a)** Positive control (FLK cell line constitutively infected with BLV). **(b)** Breast cancer tissue. Mixed type carcinoma. Sample negative for BLV. No brown staining in mammary epithelial cells. **(c)** Normal breast tissue. Mammary epithelial cells (lobules) observed within defined areas with brown staining. BLV positive (representative sample). **(d)** Breast cancer tissue. Poorly differentiated ductal carcinoma. Irregular structures visualised. Mammary epithelial cells with brown staining. BLV positive (representative sample). Arrows for **(c)** and **(d)** indicate the BLV genome by incorporating digoxigenin-labelled uracil in PCR products. Images visualised at 10 and 40X on a Nikon Eclipse E200 optical microscope. Images are representative examples of *in situ* PCR results. Colour balance and brightness were adjusted similarly for all the pictures.

S1 Table. Detailed results of the presence of BLV and diagnoses in the cohort of patients.

SAMPLE ID	AGE	PATHOLOGY	DIAGNOSIS	BLV DETECTION					BLV (+) **
				Nested PCR: Breast tissue	Nested PCR: Blood	IS PCR	nPCR + IS PCR	IHC (p24)	
1 HM	76	M	Invasive poorly differentiated carcinoma	-	-	-		-	-
2 HM	52	B	Sclerosis adenosis	-	-	ND		ND	-
3 HM	54	B	Hyperplasia without atypia	-	-	ND		-	-
4 HM	72	B	Hyperplasia without atypia	-	-	-		-	-
5 HM	20	B	Fibroadenoma	-	-	ND		ND	-
6 HM	82	M	Invasive ductal carcinoma	-	-	ND		ND	-
7 HM	73	B	Hyperplasia without atypia	-	-	ND		-	-
8 HM	63	B	Papillary lesion	+	+	-		-	+
9 HM	24	PM	Atypical hyperplasia	+	+	+	+	-	+
10 HM	81	PM	Papillary lesion with atypia	+	+	ND		-	+
11 HM	61	M	Invasive lobular carcinoma	+	+	-		-	+
12 HM	34	B	Fibrocystic change	+	+	+	+	+	+
13 HM	20	B	Fibroadenoma	-	-	-		-	-
14 HM	36	M	Invasive ductal carcinoma	-	-	-		-	-
15 HM	62	M	Invasive lobular carcinoma	-	-	ND		ND	-
16 HM	61	PM	In situ carcinoma	-	-	-		-	-
17 HM	68	M	Sarcoma	-	-	-		-	-
18 HM	41	B	Fibroadenoma	-	-	-		-	-
19 HM	24	B	Fibroadenoma	+	+	+	+	-	+
20 HM	47	B	Sclerosis adenosis	-	-	-		-	-
21 HM	51	B	Fibroadenoma	-	-	-		-	-
22 HM	65	B	Papillary lesion	-	-	-		-	-
23 HM	67	PM	In situ carcinoma	-	-	ND		ND	-
24 HM	69	M	Invasive ductal carcinoma	-	-	-		-	-
25 HM	74	M	Invasive ductal carcinoma	-	-	-		-	-
26 HM	81	M	Invasive ductal carcinoma	-	-	+		-	+
27 HM	69	M	Invasive lobular carcinoma	+	+	+	+	-	+
28 HM	81	M	Invasive ductal carcinoma	-	-	+		-	+
29 HM	55	M	Invasive ductal carcinoma	-	-	-		-	-
30 HM	63	M	Invasive ductal carcinoma	-	-	-		-	-
31 HM	72	M	Invasive lobular carcinoma	-	-	+		+	+
32 HM	53	B	Fibrocystic change	-	-	-		-	-

33 HM	47	M	Invasive poorly differentiated carcinoma	+	-	-		-	+
34 HM	63	B	Hyperplasia without atypia	-	-	ND		ND	-
35 HM	23	B	Fibroadenoma	-	-	-		-	-
36 HM	24	B	Fibroadenoma	+	-	+	+	-	+
37 HM	49	M	Invasive ductal carcinoma	-	-	-		-	-
38 HM	71	M	Invasive ductal carcinoma	-	-	-		-	-
39 HM	73	M	Invasive ductal carcinoma	+	+	-		+	+
40 HM	77	M	Invasive ductal carcinoma	-	-	-		-	-
41 HM	21	B	Fibroadenoma	-	-	-		-	-
42 HM	55	M	Invasive ductal carcinoma	+	-	-		+	+
43 HM	47	PM	Papillary lesion with atypia	-	-	-		-	-
44 HM	63	M	Invasive poorly differentiated carcinoma	-	-	-		-	-
45 HM	62	M	Invasive poorly differentiated carcinoma	-	-	-		-	-
46 HM	21	B	Papillary lesion	+	+	+	+	-	+
47 HM	74	M	Invasive ductal carcinoma	-	-	+		-	+
48 HM	25	B	Fibrocystic change	-	-	-		-	-
49 HM	39	B	Hyperplasia without atypia	-	-	-		-	-
50 HM	19	B	Fibroadenoma	+	+	ND		ND	+
51 HM	46	B	Fibroadenoma	-	-	ND		ND	-
52 HM	56	M	Invasive ductal carcinoma	-	-	ND		ND	-
53 HM	24	B	Fibroadenoma	-	-	-		-	-
54 HM	79	B	Sclerosis adenosis	+	+	ND		ND	+
55 HM	76	PM	In situ carcinoma	+	+	-		ND	+
56 HM	20	B	Fibroadenoma	-	-	-		-	-
57 HM	43	B	Hyperplasia without atypia	+	+	-		-	+
58 HM	78	M	Invasive ductal carcinoma	-	-	-		-	-
59 HM	52	PM	Atypical hyperplasia	-	-	-		-	-
60 HM	80	M	Invasive ductal carcinoma	+	+	-		-	+
61 HM	53	M	Invasive ductal carcinoma	-	-	-		-	-
62 HM	52	B	Sclerosis adenosis	-	ND	ND		ND	-
63 HM	25	B	Fibroadenoma	+	+	+	+	-	+
64 HM	22	B	Fibroadenoma	-	-	-		-	-
65 HM	56	PM	Atypical phyllodes tumour	-	-	-		-	-
66 HM	71	M	Invasive lobular carcinoma	+	+	+	+	-	+
67 HM	71	B	Fibroadenoma	+	+	ND		ND	+
68 HM	65	B	Simple cyst	-	-	-		-	-
69 HM	73	M	Invasive ductal carcinoma	+	+	-		-	+

70 HM	70	M	Invasive ductal carcinoma	+	+	+	+	-	+
71 HM	62	B	Simple cyst	+	+	-		+	+
72 HM	50	M	Invasive ductal carcinoma	-	-	ND		ND	-
73 HM	65	M	Invasive ductal carcinoma	+	+	ND		ND	+
74 HM	51	B	Sclerosis adenosis	-	-	-		ND	-
75 HM	35	M	Invasive ductal carcinoma	-	-	-		-	-
76 HM	75	M	Invasive ductal carcinoma	+	+	-		-	+
78 HM	18	B	Fibroadenoma	-	-	-		-	-
79 HM	62	M	Invasive lobular carcinoma	-	-	-		-	-
80 HM	22	B	Fibroadenoma	+	+	-		-	+
81 HM	76	M	Invasive ductal carcinoma	+	+	-		+	+
82 HM	68	M	Invasive lobular carcinoma	+	+	-		-	+
83 HM	56	B	Mastitis	-	-	-		-	-
84 HM	27	B	Papillary lesion	-	-	ND		ND	-
85 HM	20	B	Fibroadenoma	+	-	-		-	+
86 HM	45	B	Papillary lesion	+	+	-		+	+
87 HM	80	PM	Atypical hyperplasia	-	-	+		-	+
88 HM	70	M	Invasive ductal carcinoma	+	+	-		-	+
89 HM	68	M	Mixed type carcinoma	+	+	-		-	+
90 HM	56	M	Invasive ductal carcinoma	-	-	-		-	-
91 HM	51	B	Fibroadenoma	+	+	+	+	-	+
92 HM	77	B	Papillary lesion	-	-	ND		ND	-
93 HM	44	B	Fibroadenoma	-	-	+		-	+
94 HM	75	M	Invasive ductal carcinoma	-	-	+		-	+
95 HM	55	B	Papillary lesion	-	-	+		-	+
96 HM	84	M	Mixed type carcinoma	-	-	+		-	+
97 HM	23	B	Fibroadenoma	-	-	+		-	+
98 HM	69	B	Fibrocystic change	-	-	-		-	-
99 HM	57	M	Invasive poorly differentiated carcinoma	-	-	+		-	+
100 HM	37	B	Fibrocystic change	-	-	-		-	-
101 HM	70	B	Papillary lesion	-	-	ND		ND	-
102 HM	56	M	Invasive ductal carcinoma	-	-	-		-	-
103 HM	19	B	Fibroadenoma	-	-	+		-	+
104 HM	70	M	Invasive poorly differentiated carcinoma	+	+	-		+	+
105 HM	69	M	Invasive ductal carcinoma	+	+	ND		-	+
106 HM	46	M	Invasive ductal carcinoma	+	+	-		-	+
107 HM	52	PM	In situ carcinoma	+	+	-		-	+
108 HM	44	M	Invasive ductal carcinoma	-	-	+		-	+
109 HM	27	B	Fibroadenoma	+	+	+	+	-	+
110 HM	29	B	Fibroadenoma	+	+	-		-	+

111 HM	47	B	Fibroadenoma	-	-	-		-	-
112 HM	60	M	Invasive ductal carcinoma	+	+	-		+	+
113 HM	82	M	Invasive lobular carcinoma	-	-	+		-	+
114 HM	24	B	Fibroadenoma	-	ND	-		-	-
115 HM	50	B	Hyperplasia without atypia	-	-	ND		ND	-
116 HM	63	B	Sclerosis adenosis	+	+	-		-	+
117 HM	25	B	Fibroadenoma	+	+	-		+	+
118 HM	62	M	Invasive lobular carcinoma	+	+	+	+	+	+
119 HM	50	M	Invasive ductal carcinoma	+	+	-		-	+
120 HM	66	M	Mixed type carcinoma	+	+	+	+	+	+
121 HM	53	B	Fibroadenoma	+	+	+	+	-	+
122 HM	51	B	Fibroadenoma	-	-	+		-	+
123 HM	21	B	Fibroadenoma	-	-	+		-	+
124 HM	21	B	Fibroadenoma	-	-	-		+	-
125 HM	53	B	Papillary lesion	+	-	-		-	+
126 HM	18	B	Fibroadenoma	-	-	-		-	-
127 HM	22	B	Fibroadenoma	-	-	-		-	-
128 HM	65	M	Invasive poorly differentiated carcinoma	+	+	ND		ND	+
129 HM	76	M	Invasive ductal carcinoma	-	-	+		-	+
130 HM	58	B	Papillary lesion	+	-	-		-	+
131 HM	18	B	Fibroadenoma	-	-	-		-	-
132 HM	22	B	Fibroadenoma	-	-	+		+	+
133 HM	23	B	Fibroadenoma	+	+	-		-	+
134 HM	40	B	Fibroadenoma	-	-	+		-	+
135 HM	17	B	Fibroadenoma	+	-	+	+	-	+
136 HM	76	M	Mucinus carcinoma	-	-	-		-	-
137 HM	71	M	Invasive poorly differentiated carcinoma	-	-	+		-	+
138 HM	66	M	Invasive poorly differentiated carcinoma	-	-	ND		ND	-
139 HM	68	M	Invasive poorly differentiated carcinoma	+	+	ND		ND	+
140 HM	25	B	Fibroadenoma	+	-	+	+	-	+
141 HM	66	M	Invasive poorly differentiated carcinoma	-	ND	-		-	-
142 HM	77	M	Invasive poorly differentiated carcinoma	-	ND	-		-	-
143 HM	16	B	Fibroadenoma	+	-	-		-	+
144 HM	51	M	Invasive poorly differentiated carcinoma	+	-	-		-	+
145 HM	70	M	Invasive poorly differentiated carcinoma	+	-	-		ND	+
146 HM	45	B	Hyperplasia without atypia	-	-	-		-	-

147 HM	32	B	Fibroadenoma	+	-	-		-	+
148 HM	57	B	Simple cyst	-	-	-		-	-
149 HM	79	M	Invasive poorly differentiated carcinoma	+	-	+	+	-	+
150 HM	80	M	Invasive poorly differentiated carcinoma	+	+	-		+	+
151 HM	69	M	Invasive lobular carcinoma	+	-	-		-	+
152 HM	39	M	Malignant phyllodes tumour	+	+	-		-	+
153 HM	27	B	Fibroadenoma	-	-	-		-	-
154 HM	76	M	Invasive ductal carcinoma	+	+	+	+	-	+
155 HM	57	B	Hyperplasia without atypia	-	-	-		-	-
156 HM	48	M	Invasive poorly differentiated carcinoma	-	-	-		-	-
157 HM	48	B	Benign phyllodes tumor	+	-	+	+	-	+
158 HM	75	M	Mucinus carcinoma	+	+	+	+	-	+
159 HM	89	M	Mucinus carcinoma	+	+	+	+	-	+
160 HM	58	M	Invasive poorly differentiated carcinoma	-	-	-		-	-
161 HM	70	M	Invasive poorly differentiated carcinoma	+	+	-		-	+
162 HM	66	B	Fibrocystic change	-	-	ND		ND	-
163 HM	33	B	Fibroadenoma	-	-	-		-	-
164 HM	83	M	Invasive poorly differentiated carcinoma	-	-	-		-	-
165 HM	44	B	Hyperplasia without atypia	+	-	+	+	+	+
166 HM	45	B	Sclerosis adenosis	-	-	+		-	+
167 HM	62	M	Invasive ductal carcinoma	+	-	+	+	-	+
168 HM	52	B	Fibrocystic change	+	-	+	+	-	+
169 HM	43	B	Papillary lesion	+	-	+	+	+	+

Pathology - B: Benign, PM: Premalignant, M: Malignant.
* IS-PCR + nPCR: samples positive for both nested and in situ PCR
**BLV (+): Samples used for risk assessment. Referring to positive samples in the breast with any of the molecular techniques (nPCR/IS-PCR).
