# Supplementary material for: Risk factor for breast cancer development under exposure to bovine leukemia virus in Colombian women: A case-control study
Source: PLoS One. 2021 Sep 21;16(9):e0257492. doi: 10.1371/journal.pone.0257492 (PMC8454960; doi:10.1371/journal.pone.0257492)

Journal: PLOS ONE

Title: Risk factor for breast cancer development under exposure to Bovine Leukemia Virus in Colombian women: A case-control study

Authors: Nury N. Olaya-Galán[1,2]*, Sandra P. Salas-Cárdenas[2], Jorge L. Rodriguez-Sarmiento[3], Milcíades Ibáñez-Pinilla[4], Ricardo Monroy[4], Adriana P. Corredor-Figueroa[2], Wilson Rubiano[4], Jairo de la Peña[4], HuaMin Shen[5], Gertrude C. Buehring[5], Manuel A. Patarroyo [6,7,8], Maria F. Gutierrez[2]

Corresponding author:
nury.olaya@urosario.edu.co (NOG)

Gels and pictures

A)

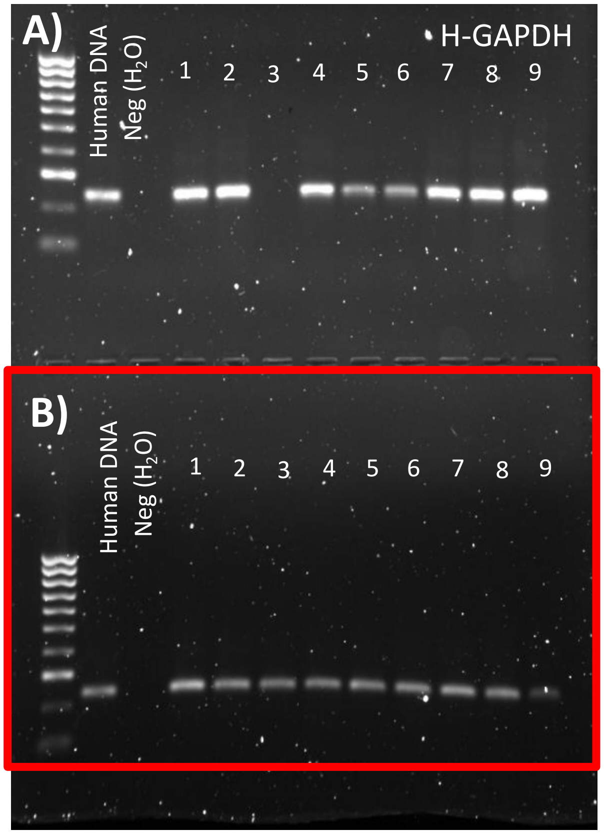

B)

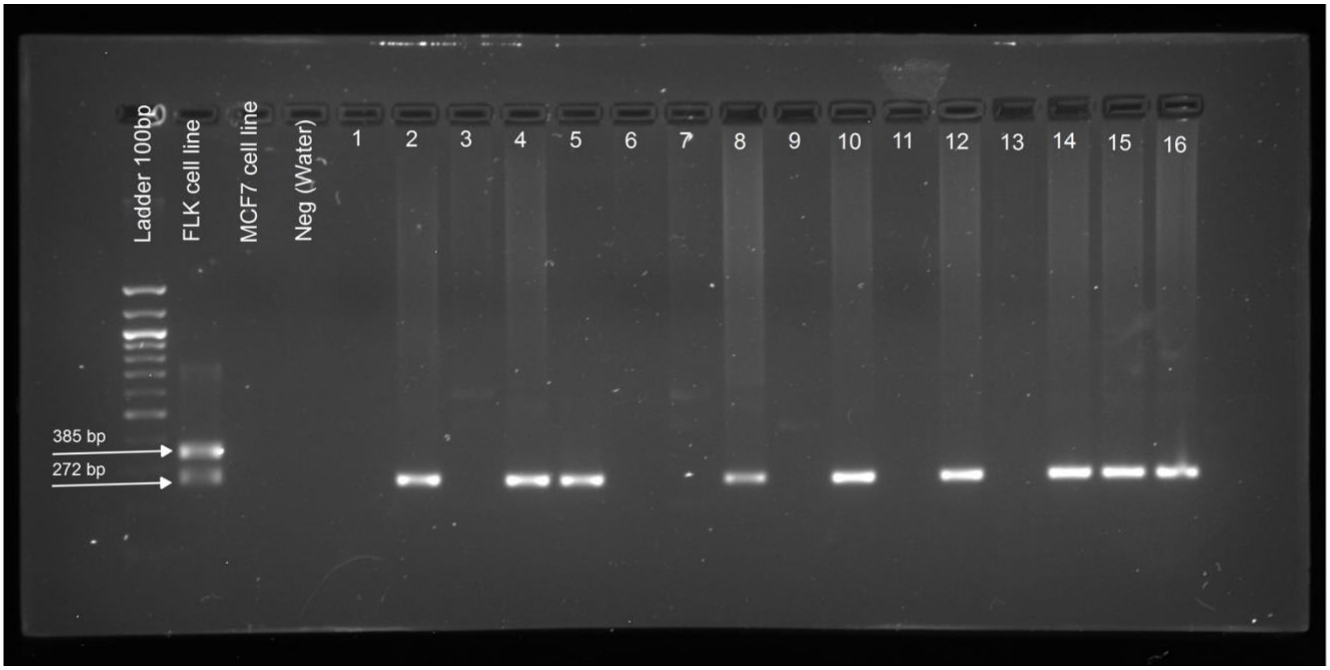

S1 Fig. Complete electrophoresis gels for human GAPDH (A) and *gag* gene (B). 1.5% Agarose gels, stained with ethidium bromide. For GAPDH, two independent experiments are shown. Results shown in the main manuscript belong to panel B . No changes in terms of color and contrast were performed to the gels. In the case of negative GAPDH samples, if extra biological material was available, DNA extraction was carried out again, otherwise sample was invalidated. Areas in red square represent the area of final composition.

COMPILED S2 FIGURE (AS SUBMITTED IN SUPPORTING INFORMATION FILE)

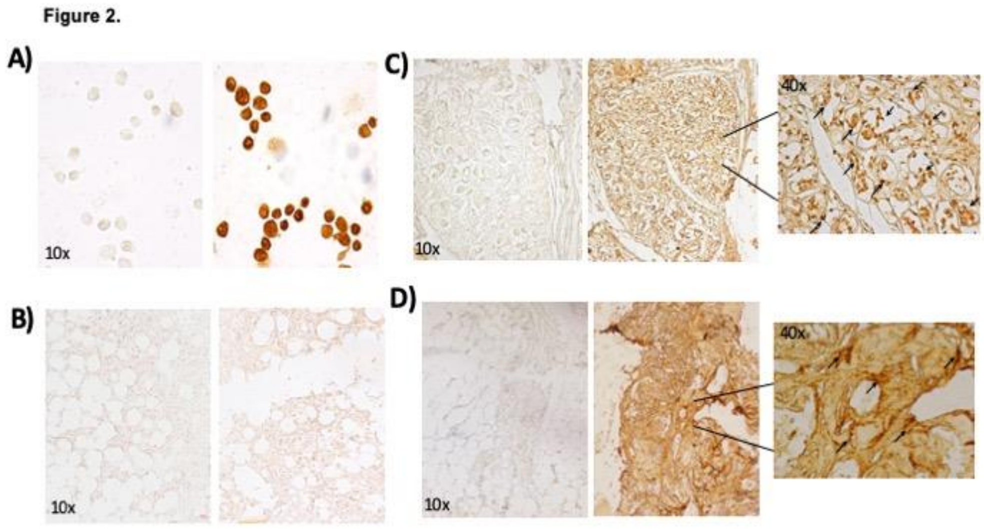

S2 Fig. *In situ* PCR of human breast tissue. *Left panels* – Adjacent tissue slice/smear for antibody control. *In situ* PCR reaction without primer and Taq polymerase control. *Right panel*s – Complete *in situ* PCR reaction. Staining was visualised by DAB reaction against anti-DIG system. **(a)** Positive control (FLK cell line constitutively infected with BLV). **(b)** Breast cancer tissue. Mixed type carcinoma. Sample negative for BLV. No brown staining in mammary epithelial cells. **(c)** Normal breast tissue. Mammary epithelial cells (lobules) observed within defined areas with brown staining. BLV positive (representative sample). **(d)** Breast cancer tissue. Poorly differentiated ductal carcinoma. Irregular structures visualised. Mammary epithelial cells with brown staining. BLV positive (representative sample). Arrows for **(c)** and **(d)** indicate the BLV genome by incorporating digoxigenin-labelled uracil in PCR products. Images visualised at 10 and 40X on a Nikon Eclipse E200 optical microscope. Images are representative examples of *in situ* PCR results. Colour balance and brightness were adjusted similarly for all the pictures.

ORIGINAL IMAGES:

S2_A FLK cell line constitutively infected with BLV – 40x

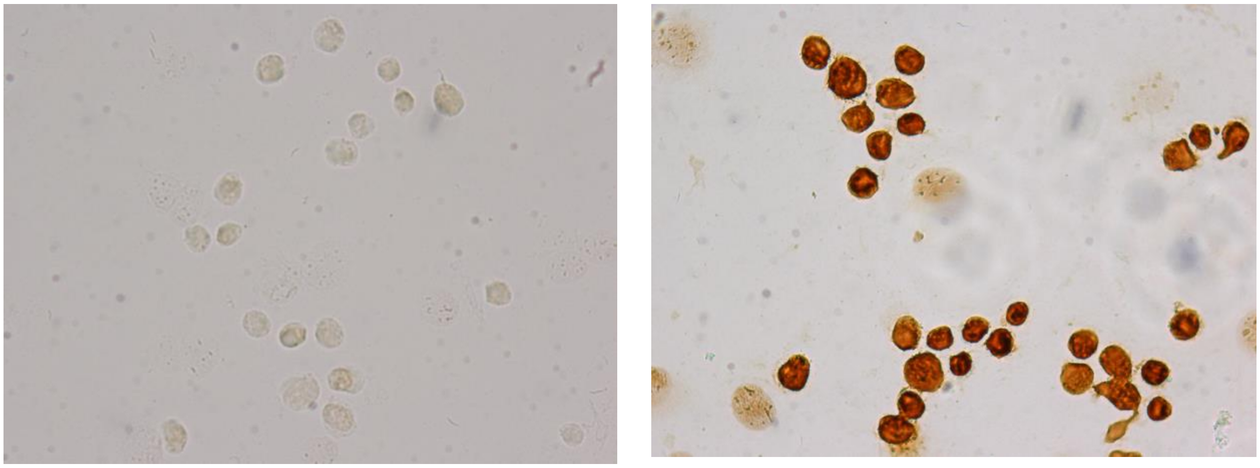

S2_B Breast cancer tissue. Mixed type carcinoma. Sample negative for BLV. No brown staining in mammary epithelial cells

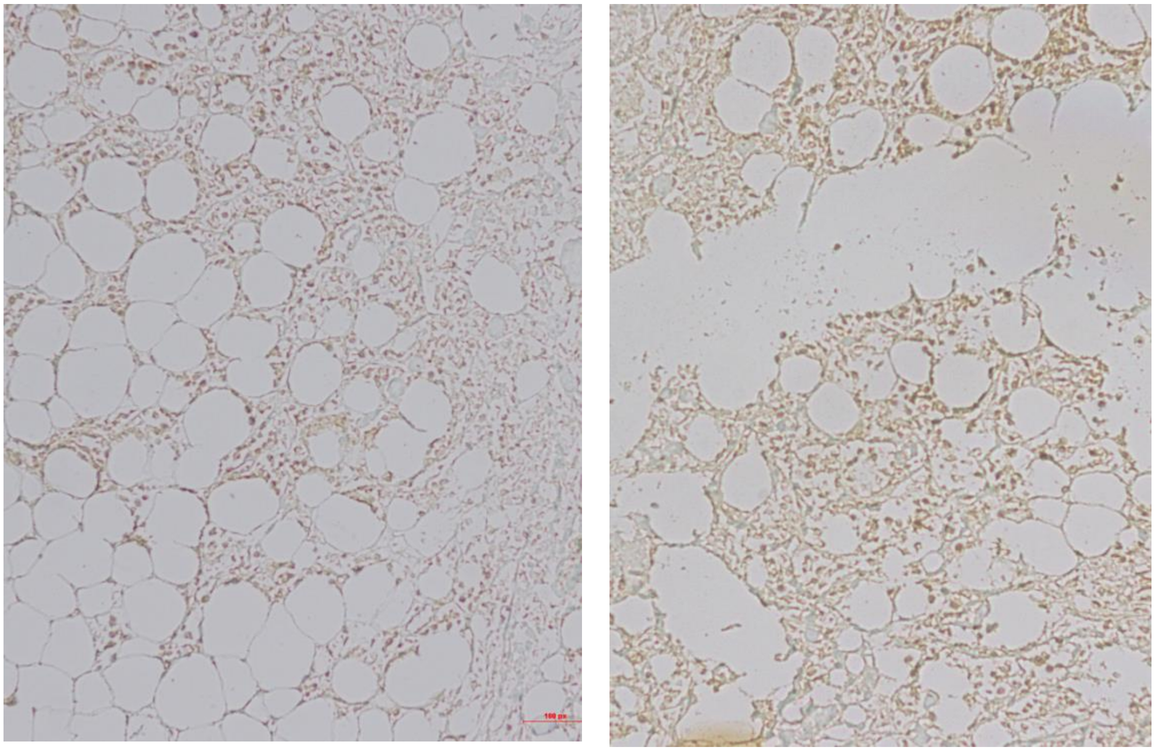

S2_C Normal breast tissue. Mammary epithelial cells (lobules) observed within defined areas with brown staining

10x

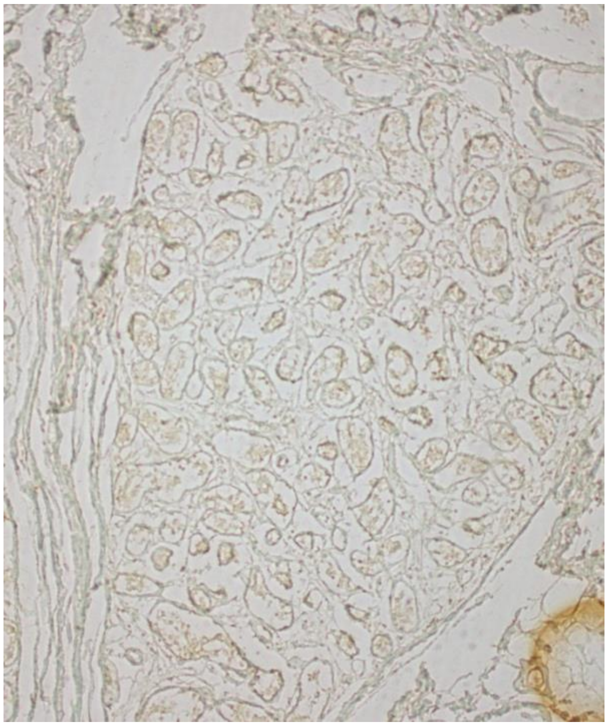

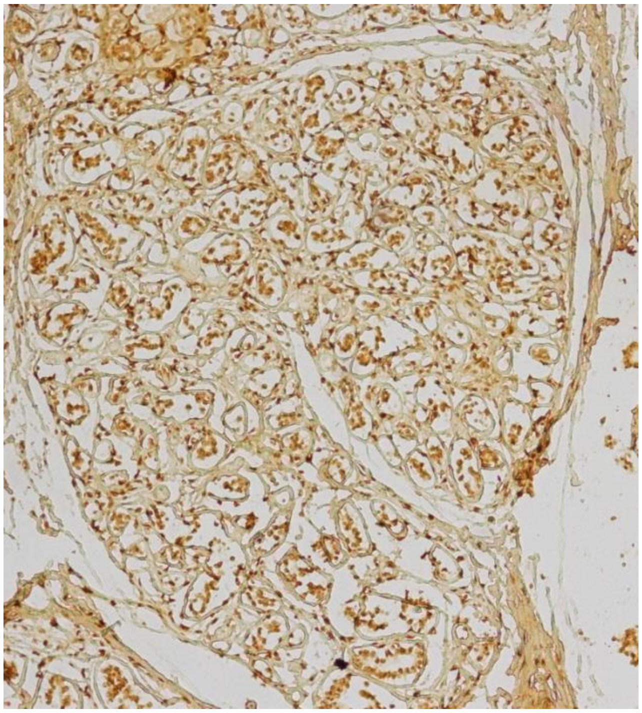

40x

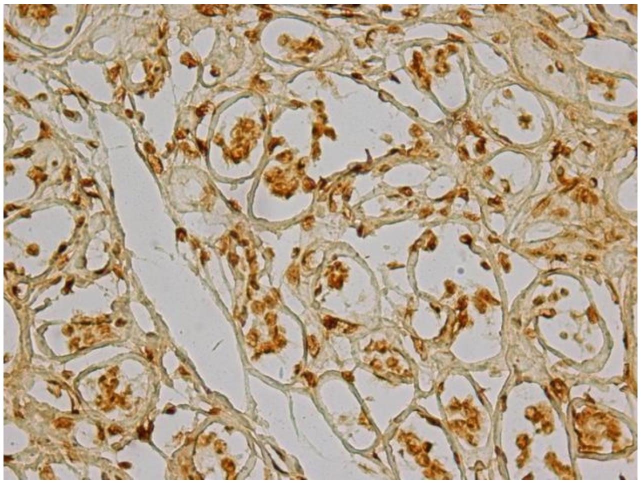

S2_D Breast cancer tissue. Poorly differentiated ductal carcinoma. Irregular structures visualised. Mammary epithelial cells with brown staining. BLV positive (representative sample)

10x

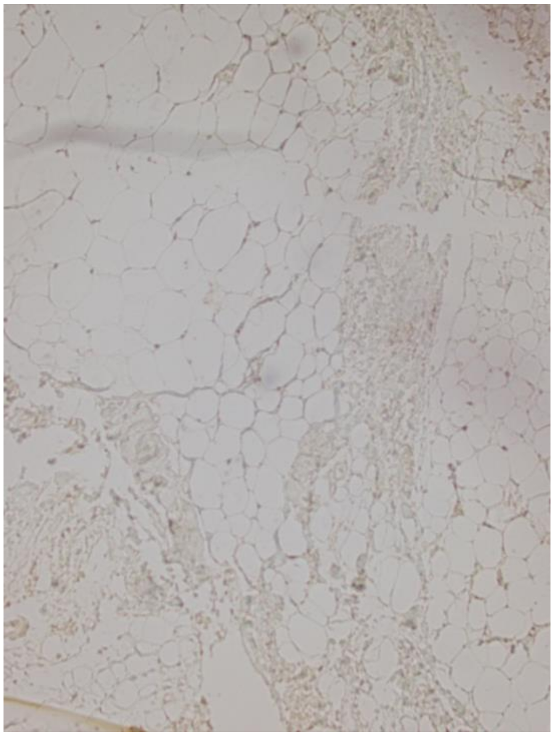

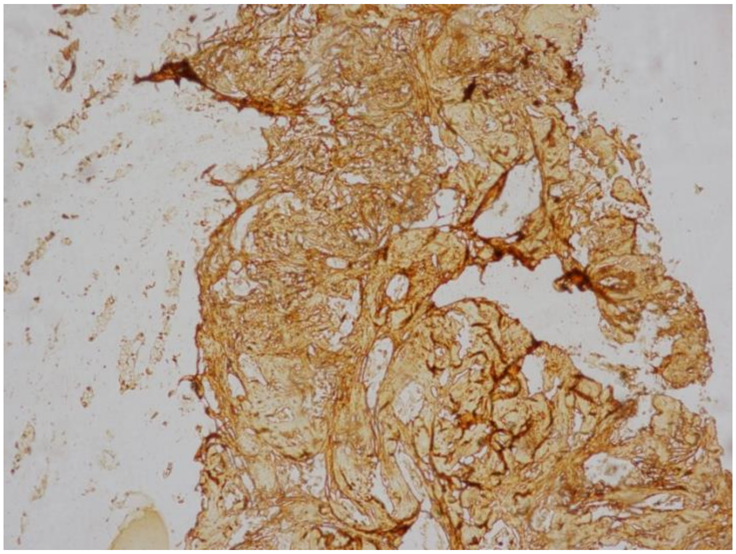

40x

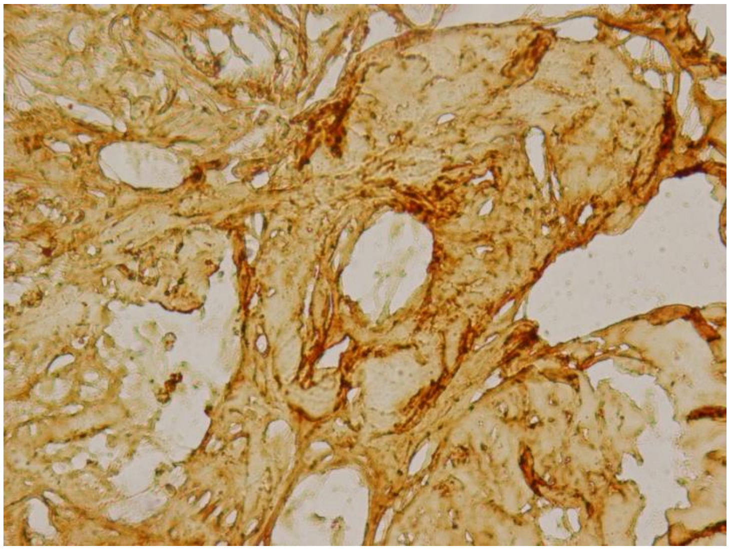

Supplement: S1 Raw images — (PDF) [file pone.0257492.s002.pdf]
